# More than two decades trapped

**DOI:** 10.1038/s41377-018-0087-9

**Published:** 2018-11-07

**Authors:** Cheng-Wei Qiu, Lei-Ming Zhou

**Affiliations:** Department of Electrical and Computer Engineering, National University of Singapore, 4 Engineering Drive 3, Singapore, 117583 Singapore

## Abstract

Optical tweezers, crowned by Nobel Prize the first time in 1990s, have widely impacted the research landscape of atom cooling, particle manipulation/sorting, and biology. After more than two decades of steady development, it received the deserving recognition once again in 2018. Unprecedented advancements across various disciplines are believed to be spurred furthermore by this important tool of optical manipulation.

Since the discovery of optical trapping force in half a century ago, it has been used and known widely as the optical tweezers^1,2^. In 1997, Steven Chu, Claude Cohen-Tannoudji, and William D. Phillips won the Nobel Prize for atom trapping and cooling by laser^3,4^. Through trapping and manipulating larger items such as bacteria and cells^5,6^, it becomes as a powerful paradigm in biological and medical science, which won Arthur Ashkin the Nobel Prize in 2018. In between those two Nobel Prizes in Physics, more than two decades have elapsed, witnessing significant progress in advanced optical micro-/nano-manipulations based on the optical tweezer concept. This “trapped” state, though not short, provides the whole community a steady yet profound opportunity to sit back, searching for groundbreaking application values. And, the optical tweezer concept eventually returns with an epic laureate, owing to its powerful and promising applications in biology.

The first observation of the mechanical effect of light force phenomenon can be traced back to 1619, i.e., more than 400 years ago, when Kepler^7,8^ observed the comet tails pointed away from the sun and captivated that it was caused by the force of light. Then, 250 years later, Maxwell^9^ crafted his theory of classical electrodynamics, in which he showed that the light carried momentum and exerted a pressure on an object if the object reflected the light. The force along with the pressure could push the object forward, in an analog to what happened in a comet tail. His theory was confirmed experimentally by Nicolas and Hull^10^.

The force caused by light is called radiative pressure since then. It was taken for granted that the radiative force can push the particle forward due to the momentum conservation law. Ashkin^1^ counter-intuitively demonstrated the gradient of the light field distribution could drag and trap the particle in the liquid with two counter-propagating laser beams. In this work, he also stated the idea of levitating atoms and molecules using resonant light with the atom transition. Ashkin and Chu^2^ further demonstrated the trapping of a dielectric particle with a single strongly focused beam and extended the trapping size range to 10 μm–25 nm, which paves the most fundamental platform of optical tweezers^2^.

Chu et al.^4^ demonstrated the trapping of atoms using laser beam and cooling of the atoms to extremely low temperature. The cooled atoms empower a plethora of applications, especially in high sensitivity metrology including atomic interferometry and atomic clock. In parallel, Ashkin continued to flourish the realm of optical tweezers. He managed to demonstrate the manipulation of single viruses and bacteria^5^, and singe cells alive^6^. The 1064 nm-wavelength infrared light has been employed, providing sufficiently large force with greatly reduced damage to the biology cell. It was followed by tremendous investigations and developments in biology science based on optical tweezers. Block et al.^11^ studied the bead movement by single kinesin molecules with optical tweezers. Yin et al.^12^ measured the force produced by a single molecule of RNA polymerase during transcription. Using an optical trapping interferometer with feedback control, Wang et al.^13^ measured the force-extension relationships of single DNA molecules. Optical tweezers can trap micrometer-/nanometer-size items with an exerted force from 100 aN to 100 pN, right in the range of the forces within cell and macromolecular systems. Thus, optical tweezers fit perfectly for investigating and even engineering various biological process, e.g., characterization of the forces of kinesin molecules^11^, probing the viscoelastic properties^13^, and doing intracellular surgery^14^. Over the past decades, the territorial boundary of optical tweezers has also been significantly extended to various other areas, including colloid and interface science^15^, microfluidic sorting by light^16^, and even quantum science and technology based on levitated opto-mechanical system^17^. The sophistication and powerfulness of the tweezer have also been greatly boosted, e.g., nanometric optical tweezers^18^ and holographic optical tweezers^19^. A schematic illustration of its historical development is shown in Fig. 1.

**Fig. 1:**
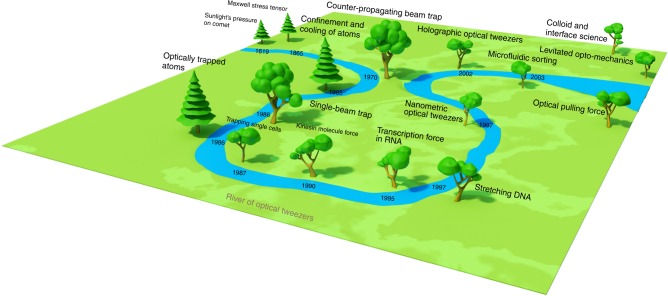
The river of optical tweezers flows on the flatland of optical force. The optical tweezers have received grand recognition and are still rapidly expanding its powerful applications in various disciplines

Although the optical tweezers have received the grand recognition of Nobel Prize twice in the past three decades, the novel physics behind the optical force still fascinate the researchers, especially when optical force meets with structured lights or materials. Novel mechanisms of optical force have been revealed. Using vortex beams, researchers reported the complex stiffness and trapping mechanism of beams with orbital angular momentum^20^. With a birefringent microparticle in vacuum, Arita et al.^21^ demonstrated 5 MHz frequency of rotation using the circularly polarized beam and proposed its application in micro-gyroscope. Using beams with angular momentum, researchers managed to trap and spin the particles^22,23^. Another unprecedentedly interesting topic is the optical pulling force—the light could pull the particle toward the light source instead of pushing away. The pulling phenomena is first studied in optical solenoid beams^24^ and afterwards the full framework for the theory of optical “tractor beam” is established^25–27^.

Apart from those exciting achievements in aforementioned areas, the optical tweezer technology rapidly expands its “contour” and synergizes with other disciplines^28–30^. For instance, it spurs the advancement in the ground-state cooling of macro-particles^17^, detection of non-Newtonian gravity^31^, detection of gravitational wave^32^, and Brownian Carnot engine^33^. Therefore, it is believed that the optical tweezers will continue to sail and explore the new edges, and return with more in near future.
